# Reduced Lung Function at Preschool Age in Survivors of Very Low Birth Weight Preterm Infants

**DOI:** 10.3389/fped.2020.577673

**Published:** 2020-09-22

**Authors:** Hung-Yang Chang, Jui-Hsing Chang, Hsin Chi, Chyong-Hsin Hsu, Chia-Ying Lin, Wai-Tim Jim, Chun-Chih Peng

**Affiliations:** ^1^Department of Pediatrics, MacKay Children's Hospital, Taipei, Taiwan; ^2^Department of Medicine, MacKay Medical College, New Taipei City, Taiwan

**Keywords:** prematurity, lung function, bronchopulmonary dysplasia, spirometry, very low birth weight

## Abstract

**Background:** Survivors of preterm birth are at risk of long-term respiratory consequences. The objective of this prospective study was to assess pulmonary function at preschool age of former very low birth weight (VLBW) preterm children.

**Methods:** Lung function of children born preterm and term controls aged 5–6 years were assessed by spirometry. The results were converted to z-scores. A questionnaire regarding respiratory symptoms was completed. Associations to gestational age (GA), birth weight (BW), bronchopulmonary dysplasia (BPD), and perinatal factors were assessed.

**Results:** In total, 85 VLBW preterm children and 29 term controls were studied. Of the preterm children, the mean GA was 28.6 ± 2.6 weeks and the mean BW was 1,047 ± 273 gm. Preterm children had significantly lower z-scores of forced expiratory volume in 1 s (FEV_1_), FEV_1_/forced vital capacity (FVC) ratio, and forced expiratory flow rate between 25–75% of FVC (FEF_25−75_), compared with term controls (−0.73 vs. 0.04, *p* = 0.002; −0.22 vs. 0.39, *p* = 0.003; −0.93 vs. 0.0, *p* < 0.001; respectively). Further segregation of the preterm group revealed significantly impaired FEV_1_, FEF_25−75_ in children at earlier gestation (≤ 28 weeks, *n* = 45), lighter at birth (≤ 1,000 g, *n* = 38), or with BPD (*n* = 55) compared with term controls (*p* < 0.05). There were significant negative relationships between the severity of BPD with FEV_1_, FVC, and FEF_25−75_ (*p* < 0.05). However, no correlation between lung function measurements and respiratory symptoms was found.

**Conclusions:** VLBW preterm infants have reduced lung function at preschool age, especially among those with younger GA, lower BW, and BPD. Additional long-term follow-up of respiratory outcomes are needed for this vulnerable population.

## Introduction

Improvements in perinatal and neonatal care have led to an increased survival for very low birth weight (VLBW; birth weight <1,501 g) preterm infants. However, morbidity among this vulnerable population remains high. The incidence of bronchopulmonary dysplasia (BPD) has shown no further decline over the past decade (1). BPD may cause irreversible damage to the lungs, which is related to long-term respiratory outcomes throughout life (1). Previous studies in the pre-surfactant era reported that airway obstruction and air trapping was common in preterm born children (1–4) particularly in those with a history of BPD (1, 2, 4, 5). Despite widespread use of antenatal steroids, surfactant therapy, and less invasive ventilation strategies, more recent cohorts suggest that significant impairments in lung structure and lung function are still major concerns (6–10). Long-term follow-up studies in preterm survivors have demonstrated that lung function or respiratory health is impaired not only at preschool age, but may also persist into childhood, adolescence, and early adulthood (5, 11–14). Indeed, some evidence revealed a decrease in lung function in preterm infants, even though they had no serious respiratory diseases during the neonatal period (15, 16). This raises the concern that prematurity itself may have a significant effect on long-term respiratory morbidity.

Although most studies have shown a reduction in lung function in preterm infants, others have shown preserved lung function during childhood (17, 18). Whether the diagnosis of BPD is a risk factor for later pulmonary morbidity has also been questioned (7, 15, 16, 19). The controversy may be explained by differences in study population, the definition of BPD, and pulmonary function methods. However, studies on respiratory function in preschool children born preterm are limited (10, 20, 21). It is not clear if any abnormalities in lung function are possible to detect earlier in life.

To better understand the importance of early pulmonary insults, we conducted this prospective, observational study to evaluate lung function in a cohort of VLBW preterm survivors at preschool age (5–6 years old). The aim of this study was to compare lung function in a group of former preterm-born children to that of a group of control children born at term with normal birth weight. We also examined the associations between gestational age (GA), birth weight (BW), and the presence of BPD in regards to lung function. Furthermore, we investigated whether the severity of BPD influences lung function.

## Materials and Methods

### Participants

This prospective cohort study was performed at MacKay Children's Hospital, which is a level III perinatal center in Northern Taiwan. Preschool children aged 5–6 years, who were born at gestational age <37 weeks and with a birth weight <1,500 g, who were followed up at our institution's premature outpatient clinic, were invited to participate in this study. Term controls (GA ≥ 37 weeks and BW > 2,500 gm g) at the same age were recruited from the outpatient clinic and wards from our hospital. Children with chromosomal abnormalities, major congenital heart or pulmonary diseases, neuromuscular diseases, significant neurodevelopmental disabilities, or inability to perform spirometry were excluded from the study. Asthma and atopy did not fall under the exclusion criteria. The study protocol was approved by the institutional review board of our institution (IRB number: 16MMHIS162e). Written informed consent was obtained from the parents or guardians of each participating child.

Data regarding the perinatal and neonatal periods for the preterm birth children were obtained from chart reviews. Small for gestational age (SGA) was defined as a birth weight below the 10th percentile according to a Taiwanese sex- and gestational age-specific reference for normal fetal growth. BPD was defined and its severity was classified according to the National Heart, Lung, and Blood Institute workshop criteria (22).

### Assessment of Lung Function

All subjects underwent a standardized clinical examination by pediatricians. Height, weight, and body mass index were measured and expressed as z-scores, adjusted for sex and age according to Taiwanese children references. At the time of lung function measurement, all participants were in stable clinical condition, without any acute respiratory tract symptoms on the day of testing, or in the previous 2 weeks. Children were asked to withhold the use of short-acting bronchodilators for 1 day before testing.

Spirometry was performed by a single experienced technician, and data were analyzed by respiratory specialists who were all blinded to the clinical details of the participant. Spirometry was performed using a spirometer (Ultima PF with RTD; MGC Diagnostics, Saint Paul, MN, USA). Measurements were performed according to the American Thoracic Society and European Respiratory Society guidelines (23). During spirometry, attempts were made to achieve at least three technically acceptable sequences. The best maneuvers among those considered technically acceptable were recorded. Flow-volume curves were obtained in order to determine the following spirometric parameters: forced expiratory volume in one second (FEV_1_), forced vital capacity (FVC), FEV_1_/FVC ratio, and forced expiratory flow between 25 and 75% of expired FVC (FEF_25−75_). Lung function data were expressed as z-scores after adjustment for height, sex, age, and race according to the Global Lung Function Initiative reference (24). Any z-scores of lung function measurements < -1.96 were considered abnormal.

### Respiratory Morbidity

Parents completed a questionnaire regarding their child's respiratory morbidity and relevant family history. The questionnaire was based on the modified Chinese version of the International Study of Asthma and Allergies in Childhood (ISAAC) questionnaire (25). Factors such as maternal smoking in pregnancy, as well as current passive smoke and pet exposure were recorded. A family history of atopy was considered positive if there was an atopic disease (atopic dermatitis, allergic rhinitis, asthma) in a first-degree relative. The questionnaire also included questions on the presence of respiratory symptoms, history of wheezing, rehospitalization due to lower respiratory tract infections (bronchiolitis, pneumonia), presence of diagnosed atopic diseases, and allergies. Regular prophylactic asthma medication included inhaled corticosteroids, beta-2-agonists, or anti-leukotriene agents within the preceding 12 months.

### Statistical Analysis

A sample size of 40 individuals was calculated to have enough power (80%) to detect at least a 0.75-point difference in the FEV_1_ z-score mean value between preterm and term control groups. Categorical data were expressed as proportions and analyzed by the chi-square test or Fisher's exact test, as appropriate. The numerical data are presented as the mean ± S.D. and analyzed by the Student's *t*-test for independent samples if normally distributed, or by the Mann–Whitney *U*-test if skewed. Differences in lung function variables between subgroups were assessed by one-way analysis of variance (ANOVA) with *post-hoc* comparisons. If the overall *P*-value from ANOVA was statistically significant, differences between subgroups were tested. The Jonckheere–Terpstra test was used to assess trends in lung function parameters across the four groups with varying BPD severity. The relationships between neonatal factors and lung function were initially assessed using Pearson's correlation test and univariate linear regressions. Neonatal factors with significant univariate correlation to function outcomes were included in the multiple linear regressions. Statistical procedures were performed with IBM SPSS version 25.0 for Windows (SPSS Inc., Chicago, IL, USA). For all analyses, a *P*-value <0.05 was considered statistically significant.

## Results

### Study Population Characteristics

At preschool age, 153 former VLBW children who attended our follow-up program were invited to join this study. Of these, 89 (58.2%) subjects consented to participate in the current study. After excluding individuals unable to perform spirometry reliably (4 subjects), 85 participants born preterm (preterm group) were enrolled in this study. The preterm group was born between April 2015 and March 2017 at a mean GA of 28.6 ± 2.7 weeks and with a mean BW of 1,039 ± 247 g. Demographic, perinatal, and neonatal characteristics for the VLBW survivors are presented in Table 1. The 85 children represented 25% of the 345 VLBW survivors born during the same period at MacKay Children's Hospital. With the exception of a lower BW (1,039 vs. 1,117 g; *p* = 0.025) and a marginally higher incidence of sepsis (24 vs. 14%, *p* = 0.055) in the study subjects, there were no significant differences in perinatal characteristics and neonatal outcomes between the VLBW study cohort and the VLBW survivors who were not included in the study (Table 1). The comparison group comprised 29 term-born controls (control group) with a mean GA of 38.5 ± 1.0 weeks and a mean BW of 3,030 ± 322 g. Except for GA and BW (*p* < 0.001), there were no significant differences in sex, Apgar scores, and delivery methods between the preterm and control groups.

**Table 1:** Perinatal characteristics of very low birth weight preterm infants who were enrolled in this study.

	**Enrolled group *N* = 85**	**Not enrolled Group *N* = 260**	***p*-value**
Antenatal steroids (%)	86	87	0.708
Preeclampsia (%)	23	20	0.663
PROM >18 h (%)	30	26	0.519
Cesarean section (%)	68	75	0.206
Singleton birth (%)	83	75	0.106
GA, week (mean ±*SD*)	28.6 ± 2.7	29.0 ± 2.8	0.189
BW, gm (mean ±*SD*)^*^	1,039 ± 247	1,117 ± 259	0.025
Small for gestational age (%)	36	35	0.956
Male gender (%)	46	48	0.793
Apgar score at 5 min <7 (%)	10	8	0.595
Surfactant administration (%)	40	35	0.366
Mechanical ventilation (%)	62	56	0.352
Days on mechanical ventilation (mean ±*SD*)	17.6 ± 27.4	12.0 ± 20.8	0.089
Days on CPAP (mean ±*SD*)	43.1 ± 30.7	39.5 ± 33.0	0.376
Days on oxygen (mean ±*SD*)	60.6 ± 45.0	51.8 ± 45.5	0.120
Pneumothorax (%)	2	3	0.742
BPD (%)	73	64	0.128
Sepsis (%)	24	14	0.055
PDA need treatment (%)	38	38	0.998
NEC ≥ stage 2 (%)	1	3	0.552
ROP ≥ stage 3 (%)	7	10	0.382
Severe IVH (%)	7	6	0.649
Cystic PVL (%)	4	7	0.152

*PROM, prolonged rupture of membranes; GA, gestational age; BW, birth weight; CPAP, continuous positive airway pressure; BPD, bronchopulmonary dysplasia; PDA, patent ductus arteriosus; NEC, necrotizing enterocolitis; ROP, retinopathy of prematurity; IVH, intraventricular hemorrhage; PVL, periventricular leukomalacia*.

*Data are presented as mean (SD) or number (%)*.

* *p < 0.05*.

At the time of the lung function test, the health characteristics of all participating children were summarized in Table 2. The term controls were slightly older than the preterm children because we recruited the control children after the preterm-born children had visited. Children in the preterm group were significantly shorter and lighter than term controls, but there was no difference in body mass index (BMI). Family history of atopy, passive smoke exposure, and respiratory morbidity were also similar between both groups (Table 2).

**Table 2:** Characteristics of the study population at the time of the lung function test.

	**Preterm group *N* = 85**	**Term Control group *N* = 29**	***p*-value**
Age (year) (mean ±*SD*)^*^	5.6 ± 0.6	5.9 ± 0.5	0.002
Males (%)	48	59	0.338
Height z-score (mean ±*SD*)^*^	−0.81 ± 0.92	−0.12 ± 0.94	0.001
Weight z-score (mean ±*SD*)^*^	−0.86 ± 1.02	−0.21 ± 1.17	0.005
BMI z-score (mean ±*SD*)	−0.62 ± 1.11	−0.21 ± 1.17	0.098
Family history of atopy (%)	84	69	0.138
Passive smoke exposure (%)	38	34	0.763
Pets in the home (%)	14	21	0.406
**Respiratory Morbidity**
Wheeze ever (%)	65	59	0.780
Wheeze in last 12 m (%)	53	52	0.798
Exercise induced wheeze (%)	27	31	0.579
Nocturnal cough (%)	73	78	0.760
Doctor diagnosed asthma (%)	20	15	0.937
Asthma medication (%)	12	10	0.776
Respiratory hospitalization ≥ 3 (%)	8	9	0.763

*BMI, body mass index*.

* *p < 0.05*.

### Pulmonary Function Results

#### Comparison of Lung Function in the Preterm Group and Term Controls

After adjusting for age, sex, and height, z-scores of the lung function test of the term control and the preterm group are presented in Table 3. The preterm group had significantly lower spirometric measurements (FEV_1_, *p* = 0.002; FEV_1_/FVC, *p* = 0.003; and FEF_25−75_, *p* < 0.001) than the term control children, which were further decreased in those with lower GA, lighter BW, and with a history of BPD. Pearson's correlation and linear regression analysis revealed a positive significant correlation between GA, BW, and all spirometry results. The proportion of lung function below the lower limit (defined as any of FEV_1_, FVC, FEV_1_/FVC, or FEF_25−75_ ≤ −1.96 z-scores) was significantly higher in the preterm group than in the term controls (26 vs. 10%, *p* = 0.042). Sex, delivery mode, and SGA did not affect spirometry outcomes between preterm and term groups. The analyses also revealed no significant associations of respiratory morbidities, allergy history, and lung function data between the two groups.

**Table 3:** Lung function results of the preterm group, stratified by GA, BW, and BPD, compared to term controls (expressed as z-scores).

	**FEV_**1**_**	**FVC**	**FEV_**1**_/FVC**	**FEF_**25−75**_**
**Term Control Group (*****N*** **=** **29)**
	0.04 ± 1.18	−0.12 ± 1.14	0.39 ± 0.85	0.00 ± 1.23
**Preterm Group (*****N*** **=** **85)**
	−0.73 ± 1.12^**^	−0.60 ± 1.20	−0.22 ± 0.16^**^	−0.93 ± 1.14^***^
**Preterm, GA Subgroup**
≤ 28 weeks (*N* = 45)	−0.89 ± 1.08^**^	−0.64 ± 1.20	−0.46 ± 1.32^**^^,^ ^#^	−1.32 ± 1.13^***^^,^ *^##^*
29–36 weeks (*N* = 40)	−0.55 ± 1.16^*^	−0.56 ± 1.22	0.03 ± 0.89	−0.49 ± 1.01
**Preterm, BW Subgroup**
≤ 1,000 g (*N* = 38)	−1.17 ± 0.98^***^^,^ ^αα^	−1.00 ± 1.08^**^^,^ ^αα^	−0.30 ± 1.28^*^	−1.26 ± 1.19^***^^,^ ^α^
1,001–1,500 g (*N* = 47)	−0.38 ± 1.12	−0.29 ± 1.22	−0.17 ± 1.06^*^	−0.66 ± 1.04^*^
**Preterm, BPD Subgroup**
BPD (*N* = 55)	−0.96 ± 1.08^***^^,^ ^μ^	−0.78 ± 1.23^*^	−0.32 ± 1.29^**^	−1.18 ± 1.14^***^^,^ ^μμ^
No BPD (*N* = 30)	−0.31 ± 1.10	−0.29 ± 1.10	−0.07 ± 0.84	−0.46 ± 1.01

*GA, gestational age; BW, birth weight; BPD, bronchopulmonary dysplasia; FEV_1_, forced expiratory volume in 1 s; FVC, forced vital capacity; FEF_25−75_, forced expiratory flow between 25 and 75% of expired FVC*.

*Data were adjusted for age, sex, and height and are expressed as mean ± SD*.

* *p < 0.05 vs. controls*,

** *p < 0.01 vs. controls*,

*** *p < 0.001 vs. controls*.

# *p < 0.05 vs. GA 29–36 wk*,

## *p < 0.01 vs. GA 29–36 wk*.

α *p < 0.05 vs. BW > 1,000 g*,

αα *p < 0.01 vs. BW > 1,000 g*.

μ *p < 0.05 vs. no BPD*,

μμ *p < 0.01 vs. no BPD*.

#### GA Subgroup and Lung Function (Table 3)

Z-scores of FEV_1_, FEV_1_/FVC, and FEF_25−75_ were significantly lower in children born ≤ 28 weeks of gestation than in term controls (*P* = 0.003, 0.004, and < 0.001, respectively), but not for FVC (*P* = 0.07). Children in the ≤ 28 weeks subgroup also had significantly impaired FEV_1_/FVC and FEF_25−75_ compared to children in the 29–36 weeks subgroup (*P* = 0.008 and < 0.001, respectively). Except for FEV_1_ (*p* = 0.04), there were no differences between the 29–36 weeks subgroup and term controls. The proportion of children with lung function below the lower limit of normal in the ≤ 28 weeks subgroup (36%) was significantly higher than in the 29–36 weeks subgroup (15%) and term controls (10%) (*P* = 0.008 and 0.028, respectively).

#### BW Subgroup and Lung Function (Table 3)

All spirometry values were significantly lower in children with a BW ≤ 1,000 gm (ELBW) than in term controls (FEV_1_, *p* < 0.001; FVC, *p* = 0.002; FEV_1_/FVC, *p* = 0.01; FEF_25−75_, *P* < 0.001). Children in the ELBW subgroup also had significantly impaired FEV_1_, FVC, and FEF_25−75_ than children in the 1,001–1,500 g subgroup (*P* = 0.001, 0.006, and 0.014, respectively). The proportion of lung function below the lower limit of normal in the ≤ 1,000 g subgroup (42%) was significantly higher than in the 1,001–1,500 g subgroup (13%) and term controls (10%) (*P* = 0.003 and 0.002, respectively).

#### BPD, Severity of BPD, and Lung Function (Table 3 and Figure 1)

Among children in the preterm group, 55 (65%) had BPD in the neonatal period. All spirometry values were significantly lower in preterm children with BPD than in term controls (Table 3, FEV_1_, *p* < 0.001; FVC, *p* = 0.02; FEV_1_/FVC, *p* = 0.004; FEF_25−75_, *P* < 0.001). The preterm children in the BPD subgroup also had significantly lower FEV1 and FEF_25−75_ than the preterm no BPD subgroup (Table 3, *P* = 0.01, 0.005, respectively).

**Figure 1:**
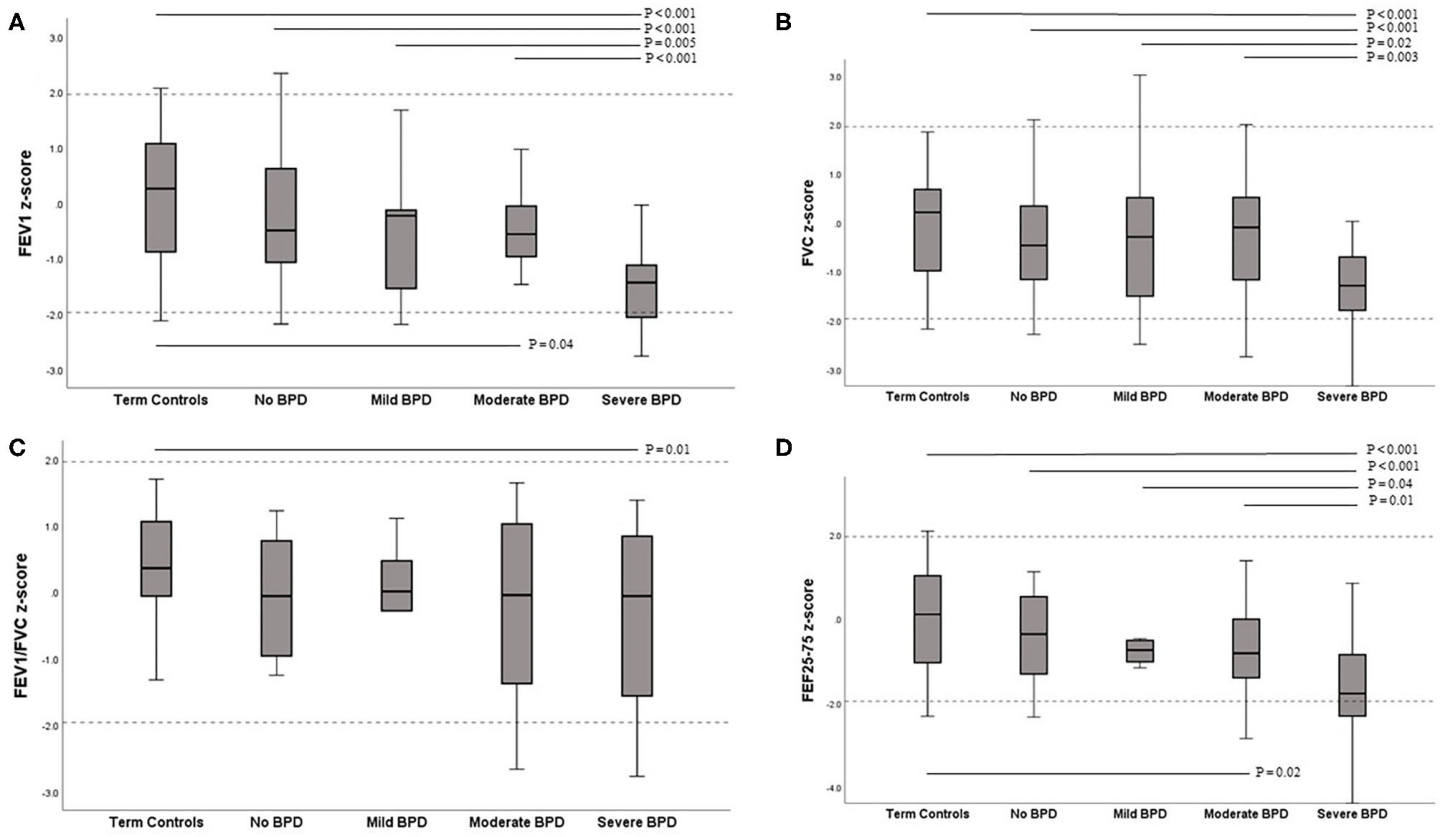
Box-plots of z-scores for lung function variables contrasted between term controls and BPD severity subgroups. **(A)** FEV_1_ **(B)** FVC **(C)** FEV_1_/FVC **(D)** FEF_25−75_. The solid line within the box is the median (50th percentile), the margins of the box are the interquartile range (IQR; 25–75th centiles), and the range of the data up to 1.5 times the IQR is shown by the error bars. Dashed lines represent ±1.96 SDs from the mean (limits of normal). FEV_1_, forced expiratory volume in 1 s; FVC, forced vital capacity; FEF_25−75_, forced expiratory flow between 25 and 75% of expired FVC.

Lung function results according to the BPD severity classification are presented in Figure 1. Among the preterm group with BPD, nine were classified as mild, 20 as moderate, and 26 as severe BPD. The most marked reductions were seen in children in the severe BPD subgroup, in whom all lung function results were significantly lower than in term controls (all had *p* < 0.001). A significant difference in lung function, which was lower in children with severe BPD compared to the moderate, mild, and no BPD subgroup was also seen for FEV_1_ (*P* < 0.001, 0.005, and < 0.001, respectively), FVC (*p* = 0.003, 0.017, and < 0.001, respectively), FEF_25−75_ (*p* = 0.014, 0.035, and < 0.001, respectively). Preterm children in the moderate BPD subgroup also had significantly lower FEV_1_ and FEF_25−75_ than children in term controls (*P* = 0.038 and *P* = 0.021, respectively). There were no differences in any lung function variables between the moderate BPD, mild BPD, and no BPD subgroups. In the preterm group, there was a significant trend toward a decreased value of FEV_1_, FVC, and FEF_25−75_ as the grade of BPD severity increased (Figure 1, Jonckheere-Terpstra trend test *p* < 0.001, 0.003, and < 0.001, respectively). The proportion of lung function below the lower limit of normal in the severe BPD subgroup (50%) was significantly higher than that in the moderate BPD (20%), no BPD (10%), and term controls (10%) (*P* = 0.03, 0.001, and 0.001, respectively).

#### Perinatal Factors and Lung Function in the Preterm Group

Associations between lung function and the factors summarized in Table 1 were investigated. Univariate analysis showed lower GA and lighter BW, longer duration of mechanical ventilation and oxygen treatment, and a history of BPD were significantly associated with lower FEV_1_, FVC, and FEF_25−75_ z-scores. However, in multivariate regression analysis using BW, the duration of mechanical ventilation, and BPD as covariates, BW was an independent variable that only moderately correlated with FEV_1_, FVC, and FEF_25−75_ (*r* = 0.49, *P* = 0.003; *r* = 0.39, *P* = 0.04; and *r* = 0.49, *P* = 0.008, respectively).

## Discussion

This study provides the most up-to-date lung function results in a cohort of VLBW preterm children, born in the era of the use of surfactant and non-invasive respiratory support strategies. Nearly 90% of our preterm children had received antenatal corticosteroids. Despite widespread use of these modern treatments, the results of the present study show that lung function was significantly impaired at preschool age in former VLBW preterm infants when compared with term controls. Although the mean values of all spirometry results in both groups were in the normal range, the preterm children still had a significantly high proportion of abnormal lung function. Furthermore, preterm children with earlier GA, lower BW, and a diagnosis of BPD in early life was associated with a marked reduction in spirometry.

Our findings are in accordance with previous reports from most contemporary cohorts (6–10). Children born preterm are at high risk for large airway obstructions, as assessed by FEV_1_ (8, 19, 26). Reduction in small airways patency, as assessed by FEF_25−75_, also continues to be reported throughout various school ages (6, 19, 26, 27). The persistence of airway obstruction, airflow limitation, and air trapping in these children is multifactorial in nature, potentially reflecting the impact of preterm birth *per se*. The vulnerability of the immature lung is susceptible to early life adverse exposures, such as volutrauma, atelectrauma, barotrauma, biotrauma, and oxygen toxicity (1). Preterm itself, together with these injuries, might result in impaired lung growth, with smaller airways and decreased lung volume (22). In our study, the difference in the mean values of the z-scores of FEV_1_, FEV_1_/FVC, and FEF_25−75_ between preterm and term controls was −0.77, −0.61, and −0.93, respectively. These differences were even greater in the GA ≤ 28 weeks subgroup than in the controls in the current study. A recent meta-analysis also confirmed that younger gestational age had a lower FEV_1_, FEV_1_/FVC, and FEF_75_ in childhood (1, 28). Together with our data, these studies indicate that the degree of preterm birth has an effect on later lung function.

In agreement with previous studies, low BW is associated with decreased lung function in preterm children (3, 7, 11, 27, 29). A previous meta-analysis and studies from longitudinal birth cohorts reported independent effects and strong positive associations of BW with FEV_1_ and FEF_25−75_ (30). Low BW with relatively small airways could result in a reduction in expiratory flow, reflected by lower lung function values. However, low BW includes both immature infants with appropriate growth for gestation, and intrauterine growth restriction (IUGR), who may be physiologically mature at birth. The mechanisms that lead to lung function deficits are different in these two conditions. However, we did not find the association between IUGR and lower lung function in the present study.

A recent meta-analysis by Ronkainen et al. confirmed that school-aged children born preterm with BPD have impaired pulmonary function measurements (8). Simpson et al. showed a reduction of −1.06 z-score FEV_1_ for the BPD group, which is comparable to our study (29). In contrast, preterm children without BPD have similar lung function results compared to term controls. Long-term follow-up of BPD survivors has also demonstrated the persistence of airway obstructions and air trapping into childhood, adolescence, and adulthood (1, 4, 11, 26, 31–33). However, conflicts exist regarding BPD severity classification and long-term lung function (8, 10, 34). Brostrom et al. reported that the impairment of lung function was most pronounced in severe BPD (15). Hirata et al. also showed that the classification of BPD severity was useful for predicting the impairment of long-term lung function (35). Consistent with these reports, we found that the severity of BPD had a significant negative association with FEV_1_, FVC, and FEF_25−75_. However, flow characteristics in mild BPD were not significantly different from those of preterm children without BPD and term controls. It might be speculated that mild BPD in preterm infants may not result in long-term respiratory consequences. However, these results should be interpreted cautiously, given the small number of mild cases in the present study.

The duration of oxygen supplementation and mechanical ventilation have been associated with diminished lung function in many studies (7, 9, 29, 32, 36). However, they might be more closely related to the degree of prematurity itself. Many other perinatal factors have been identified as important factors predicting adverse lung function in childhood. These include but are not limited to antenatal steroids, maternal nutrition, maternal pregnancy disorders (hypertensive disorders, gestational diabetes, chorioamnionitis), parental smoking, socioeconomic status, IUGR, sex, and neonatal caffeine administration (12, 37, 38). However, we did not find an association between these factors and lung function. Neither family atopic history nor exposure to pets was associated with any of the lung function measurements in the present study.

Preterm birth or low birth weight is associated with an increased risk of respiratory morbidities, especially in those with BPD (19, 28, 39). Asthma or recurrent wheezing, use of asthma medications, and hospitalization for respiratory infections are more prevalent later in life in those born preterm than term controls (9, 21, 40). Preterm children with these respiratory morbidities are likely to negatively influence lifetime lung function (26). However, we found no significant difference in respiratory morbidities between the preterm group and term controls. Furthermore, no correlation was identified between pulmonary function test results and the presence of respiratory morbidities in the present study. It is still unclear if the deficits in pulmonary function are translated into increased respiratory symptoms. Therefore, further studies are needed to disentangle the direction of causality.

This study has several limitations. The main limitation is the large loss to follow-up rate in our cohort of survivors. Selection bias may already exist due to sepsis being marginally higher in the enrolled patients since sepsis has been recognized as a risk factor for BPD (41, 42). Except for BW and sepsis, given that perinatal factors were similar between the VLBW preterm children who were included in the present study and those who were not, we are confident that the results reported represent the total cohort of survivors. Second, the questionnaire reported by parents may not be accurate, and they may overestimate the incidence of wheeze symptoms and asthma. Third, the lack of a true normal control population of healthy children is another concern. Parents may have been more motivated to participate in this study than others if their children had respiratory symptoms. Although the term controls were significantly older than preterm group, the z-scores calculations of each lung function parameter were already adjusted for age. Otherwise, the mean spirometry data in our control group exhibited approximately 0.0 z-scores, indicating that they can be used as a reference group. However, older participants in the term controls also raised the concern that they may perform better at spirometry than preterm children. Although it is not easy to perform spirometry in this age, our technician trained these children well and conducted the test according to the guidelines. Furthermore, we only performed baseline lung function tests; after exercise or bronchodilator administration tests were not assessed in this study. Further studies with adequate power are also needed to identify which perinatal factors are related to long-term adverse respiratory and lung function outcomes.

In conclusion, preterm-born children at preschool age had significantly reduced lung function compared with the term controls. Stratified analyses showed the worst results in preterm children with younger GA, lower BW, and with BPD. The deficits were particularly evident in pulmonary flow measurements, reflective of increased airway obstructions. The severity of BPD can be a predictor of lung function impairment, as severe BPD had the worst lung function outcomes. However, preterm children without BPD or with mild BPD had similar lung functions to term controls. No correlation between lung function and respiratory symptoms in this study needs further investigation. Our study suggests that adverse effects of preterm birth on lung function can be detected in early childhood. It has been proposed that preterm birth may predispose individuals to chronic obstructive pulmonary disease. With increasing survival of preterm births, efforts should be made to identify subgroups at higher risk of impaired lung function in later life. Long-term follow-up of lung function in VLBW preterm infants is warranted.

## Data Availability Statement

The original contributions presented in the study are included in the article/supplementary material, further inquiries can be directed to the corresponding author/s.

## Ethics Statement

The studies involving human participants were reviewed and approved by Institutional Review Board of MacKay Memorial Hospital (IRB number: 16MMHIS162e). Written informed consent to participate in this study was provided by the participants' legal guardian/next of kin.

## Author Contributions

H-YC, HC, and C-CP designed the project of this study. J-HC, C-HH, and C-YL performed participant recruitment. H-YC and W-TJ performed data collection and statistics. H-YC, J-HC, and C-CP prepared the draft of the manuscript. All authors approved the final manuscript as submitted and agree to be accountable for all aspects of the work.

## Conflict of Interest

The authors declare that the research was conducted in the absence of any commercial or financial relationships that could be construed as a potential conflict of interest.
